# Real-time PCR in detection and quantitation of *Leishmania donovani* for the diagnosis of Visceral Leishmaniasis patients and the monitoring of their response to treatment

**DOI:** 10.1371/journal.pone.0185606

**Published:** 2017-09-28

**Authors:** Faria Hossain, Prakash Ghosh, Md. Anik Ashfaq Khan, Malcolm S. Duthie, Aarthy C. Vallur, Alessandro Picone, Randall F. Howard, Steven G. Reed, Dinesh Mondal

**Affiliations:** 1 Laboratory of Emerging Infections and Parasitology, Nutrition and Clinical science division, International Centre for Diarrhoeal Disease Research, Dhaka, Bangladesh; 2 Infectious Disease Research Institute, Seattle, Washington, United States of America; 3 InBios International Inc, Seattle, Washington, United States of America; Taibah University, SAUDI ARABIA

## Abstract

Sustained elimination of Visceral Leishmaniasis (VL) requires the reduction and control of parasite reservoirs to minimize the transmission of *Leishmania donovani* infection. A simple, reproducible and definitive diagnostic procedure is therefore indispensable for the early and accurate detection of parasites in VL, Relapsed VL (RVL) and Post Kala-azar Dermal Leishmaniasis (PKDL) patients, all of whom are potential reservoirs of *Leishmania* parasites. To overcome the limitations of current diagnostic approaches, a novel quantitative real-time polymerase chain reaction (qPCR) method based on Taqman chemistry was devised for the detection and quantification of *L*. *donovani* in blood and skin. The diagnostic efficacy was evaluated using archived peripheral blood buffy coat DNA from 40 VL, 40 PKDL, 10 RVL, 20 cured VL, and 40 cured PKDL along with 10 tuberculosis (TB) cases and 80 healthy endemic controls. Results were compared to those obtained using a *Leishmania*-specific nested PCR (Ln-PCR). The real time PCR assay was 100% (95% CI, 91.19–100%) sensitive in detecting parasite genomes in VL and RVL samples and 85.0% (95% CI, 70.16–94.29%) sensitive for PKDL samples. In contrast, the sensitivity of Ln-PCR was 77.5% (95% CI, 61.55–89.16%) for VL samples, 100% (95%CI, 69.15–100%) for RVL samples, and 52.5% (95% CI, 36.13–68.49%) for PKDL samples. There was significant discordance between the two methods with the overall sensitivity of the qPCR assay being considerably higher than Ln-PCR. None of the assay detected *L*. *donovani* DNA in buffy coats from cured VL cases, and reduced infectious burdens were demonstrated in cured PKDL cases who remained positive in 7.5% (3/40) and 2.5% (1/40) cases by real-time PCR and Ln-PCR, respectively. Both assays were 100% (95% CI, 95.98–100) specific with no positive signals in either endemic healthy control or TB samples. The real time PCR assay we developed offers a molecular tool for accurate detection of circulating *L*. *donovani* parasites in VL, PKDL and RVL patients, as well as being capable of assessing response to treatment. As such, this real time PCR assay represents an important contribution in efforts to eliminate VL.

## Introduction

Visceral leishmaniasis (VL) is a systemic disease and the most severe clinical form of the vector borne disease complex Leishmaniasis. During VL, *Leishmania* parasites infect predominantly macrophages of the visceral organs, usually spleen, liver and bone marrow [1]. Each year approximately 300,000 VL cases occur globally, with over 20,000 case fatalities [2]. VL is endemic in large areas of the tropics, subtropics and the Mediterranean Basin, where it is variably also known as Kala-azar, Black fever or Dumdum fever. The causative agent of VL in East Africa and the Indian subcontinent is *L*. *donovani*, whereas in Europe, North Africa and Latin America it is *L*. *infantum* [3]. In the Indian sub-continent, the disease is anthroponotic and is transmitted exclusively by the sandfly vector *Phlebotomus argentipes* [4,5].

Long-term low-grade fever, hepatosplenomegaly, weight loss, pancytopenia, and polyclonal (IgG and IgM) hypergammaglobulinemia are among the common clinical signs and symptoms of VL. Delayed diagnosis and inconvenient treatment options increase the fatality rate [3]. Post-kala azar dermal leishmaniasis (PKDL) is a convoluted dermatosis that develops as a sequelae after an apparently successful treatment of VL. In East Africa >50% of VL patients develop mostly self-healing papular or nodular lesions within months of cure. In the Indian sub-continent, 10–20% of treated VL patients develop prevalently maculo-papular lesions within 2 to 10 years of treatment and require further intervention [6]. Surprisingly, 10% of PKDL patients in Bangladesh do not have any previous history of VL, indicating that these individuals probably had a prior sub-clinical *L*. *donovani* infection that went undetected [7].

60% of the global burden of VL is carried by three countries- Bangladesh, India, and Nepal. [8]. With a goal of decreasing the incidence to less than one per 10,000 population at upazila level in Bangladesh, the sub-district (block PHC) level in India, and the district level in Bhutan and Nepal, these four countries signed a memorandum of Elimination of Kala-azar by 2015 [8]. Since then, a number of ancillary projects have been launched to accelerate the progress of the program concerning different sectors including vector control, active case detection, case management & treatment and pharmacovigilance [9]. The program subsequently extended its activity until 2017 [10]. The strategies implemented within this elimination program have reduced active VL cases considerably in the past few years in hyper-endemic foci of Bangladesh [9]. Eradication is impeded, however, by the increase of relapsed VL cases (RVL), the development of PKDL, and the presence of a large population who are asymptomatically infected with *L*. *donovani*. For example, it has been reported that recurrence of VL occurs in 3.7% of treated patients within 24 months after treatment [9] and as many as 5% of the endemic population carries asymptomatic *L*. *donovani* infection without any overt sign of disease [11]. All of these groups represent potential reservoirs of parasites [12], and the PKDL and asymptomatic infected populations directly account for inter-epidemic disease transmission [6]. Therefore, proper assessment of cure (parasite clearance), along with the early detection of PKDL and asymptomatic *L*. *donovani* infection, has been emphasized necessary for the success of the elimination program [13][6].

Current diagnostic algorithms for VL primarily rely on recognizing the clinical manifestation of disease, with support from parasitological or immunological approaches [14]. The gold standard for diagnosis is the direct microscopic demonstration of *L*. *donovani* amastigotes in a splenic, bone marrow or lymph node aspirate for VL [3] or within skin biopsy or slit skin aspirate smears for PKDL [15]. While these methods are highly specific, sensitivity varies depending on the type of specimen, and results frequently suffer in terms of reproducibility [3]. The sensitivity of microscopy is consistently poor for skin biopsy of PKDL patients because of the low parasite numbers, particularly in macular lesions, that often lead to false negative results and misdiagnosis [15–17]. Furthermore, organ biopsy, the current sample collection method from tissue, can be prone to fatal hemorrhage or secondary infection such that the technical expertise required makes microscopy incompatible with routine or widespread diagnostic use [3][16].

Among various indirect immunodiagnostic markers that detect circulating antibodies, the direct agglutination test (DAT) based on whole promastigotes of *L*. *donovani* and the immunochromatographic test (ICT) based on rK39, are routinely used in the primary diagnosis of VL because of their relative simplicity and applicability within field settings [18]. One of the major limitations of these methods is, however, their inability to differentiate active from relapsed VL cases because the antigen-specific antibodies persists long after VL cure [19]. They are therefore not compatible with monitoring treatment outcomes nor can they be used to diagnose PKDL/RVL cases [15]. Thus, establishing a sensitive laboratory test that can be used in the routine diagnosis of specific clinical states, and in monitoring the response to treatment, is essential.

PCR-based molecular assays are definitive because they detect parasite nucleic acid and they have previously been demonstrated to be more sensitive in the diagnosis of VL, PKDL and RVL, in detecting asymptomatic *L*. *donovani* infection and in monitoring treatment efficacy [20,21]. Various PCR methods have been described that target multicopy parasite genes [18]. In PCR screening of blood samples of suspected VL cases up to 100% sensitivity have been reported [22], while 94–100% sensitivity has been reported with skin biopsy or slit skin aspirate of PKDL patients [14]. Increased sensitivity and specificity for *Leishmania* has also been reported with nested PCR (Ln-PCR) strategies, where two sets of primers were used, each specific for order Kinetoplastida and genus *Leishmania* [23–25]. More recently, due to several operational advantages and its ability to quantify parasite burden, quantitative real time PCR has emerged as the preferred technique [26]. Several SYBR-green and Taqman chemistry based single and multiplex real time PCR methods have been reported with promising sensitivity and specificity [27–31].

We previously used a standardized real time PCR assay, based on Taqman chemistry that targeted the conserved REPL-repeat region of the *Leishmania* genome, to assess the infection/disease dynamic in asymptomatic *L*. *donovani*-infected individuals [11]. In the current study, we used the assay to assess *L*. *donovani* burdens in blood samples from various VL-affected and unaffected groups. To our knowledge, this is the first study evaluating the use of real time PCR both for the potential diagnosis of VL, PKDL and RVL cases and for assessing treatment outcome of VL and PKDL cases in Bangladesh. When compared to an established *Leishmania* nested PCR, the real time PCR assay demonstrated excellent sensitivity and specificity for detection and quantification of *L*. *donovani* parasites in archived VL, PKDL and RVL samples. It also demonstrated utility for assessing response to treatment in VL and PKDL cases.

## Materials & methods

### Ethics statement

The investigation was approved by the International Centre for Diarrhoeal Disease Research, Bangladesh (icddr,b) and Rajshahi Medical College Ethical Review Committee (ERC). Each adult participant and parents/guardian of children participants provided written informed consent for the collection of samples and subsequent analysis.

### Study sites & participants

All cases (VL, PKDL and RVL) and healthy control individuals were enrolled within the Mymensingh and Rajshahi districts, Bangladesh, districts in which VL is highly endemic. Tuberculosis (TB) patients were enrolled from the National TB and Chest Hospital, Mohakhali, a region without VL. Laboratory tests were performed at International Centre for Diarrheal Disease Research, Bangladesh (icddr,b). 40 patients presenting with characteristic symptoms such as fever for more than two weeks, splenomegaly and/or hepatomegaly and positive rK39 RDT, with no history of VL, were selected as VL cases. 10 patients with previous history of VL who developed recurring symptoms after 6 months but within 1 year of treatment and who were positive by splenic smear microscopy were selected as RVL cases. 20 patients diagnosed and treated for VL in the past were included as cured VL cases. 40 patients showing characteristic skin rash and with a history of VL, who were treated for VL in the past and were positive for rK39 RDT, were selected as PKDL cases. The same patients were included as cured PKDL cases after completing treatment and disappearance of any visible skin rash. 80 clinically healthy individuals negative for rK39 RDT and no previous history of VL or PKDL were selected as endemic controls. 10 TB cases confirmed by acid fast bacilli microscopy and negative for rK39 RDT were selected as disease controls. The admission and clinical management of patients was independently undertaken by each respective hospital authority.

### Clinical specimen and preparation of template

2mL of venous blood was collected from each volunteer (except PKDL and cured PKDL) by venipuncture into an EDTA vacutainer. Blood was centrifuged at 2200 g for 20 minutes for separation of the buffy coat. A single skin punch biopsy was collected from each PKDL patient from the same lesion area before treatment and after cure and preserved in NET buffer by a previously described procedure [17]. The buffy coats and skin biopsies were transported to the icddr,b Parasitology Laboratory while maintaining a cold chain. DNA was isolated from 200 μL buffy coat or single skin punch biopsies using a QIA amp DNA tissue & blood mini kit (Qiagen, Hilden, Germany) as per the manufacturer’s instructions. Samples were collected between January 2, 2009 and July 6, 2013, and purified DNA samples were stored at -20°C until Ln-PCR and real time PCR. The study was performed between January, 2016 and July, 2016.

### *Leishmania* nested (Ln)-PCR

The Ln-PCR was performed with a Biorad’s MyCycler targeting *Leishmania* small subunit rDNA sequence by following a standard procedure [25]. Briefly, in nest-1, 2 μL of purified DNA was amplified in a final volume of 25 μL containing 12.5 μL of Bio-Rad iQ Supermix. 0.3-μmol/liter concentration of each *Kinetoplastida*-specific primers R221, R332 and 3.0 mM MgCl_2_ were added. The reactions were subjected to 40 cycles of denaturation at 94°C for 30 s, annealing at 65°C for 30 s, and extension at 72°C for 30 s. In nest-2, 50:1 diluted nest-1 PCR products in molecular grade water were used as templates. Nest-2 reaction conditions were similar to nest-1 except 1 μL template was added to a 25μl reaction volume where the concentration of each of the *Leishmania*-specific primers R223 and R333 was 0.15 μmol/L and the reaction was performed for 35 cycles. The final amplicons were visualized as a 358 bp product on a 1.5% agarose gel. In each run, molecular-grade water and DNA from healthy humans were used as negative controls, while DNA from cultured promastigotes served as a positive control. The primer sequences for Ln-PCR are illustrated in Table 1.

**Table 1:** Primer sequences of nested PCR.

Primer	Sequence	Product length (bp)
**R221**	5′-GGTTCCTTTCCTGATTTACG-3′	603
**R332**	5′-GGCCGGTAAAGGCCGAATAG-3′	
**R223**	5′-TCCCATCGCAACCTCGGTT-3′	358
**R333**	5′-AAAGCGGGCGCGGTGCTG-3′	

### Real time PCR

The real time PCR was performed by a method originally described by Vallur et al. [11]. Briefly, Taqman primers and probes were designed targeting conserved region of *Leishmania* REPL repeats (L42486.1) (Table 2) specific for *L*. *donovani* and *L*. *infantum* and synthesized by Applied Biosystems [11]. Briefly, a 20 μL reaction mix was prepared containing 5 μL template,10 μL of TaqMan® Gene Expression Master Mix (2X), 1 μL pre-ordered primer-probe mix and PCR grade water. Amplification was performed on a Biorad CFX96 icycler system with following reaction conditions: 10 min at 95° C, followed by 45 cycles of 15 seconds at 95° C and 1 min at 60° C. To quantify the parasite load of each sample, each run included one standard curve with DNA concentrations corresponding to 10,000 to 0.1 parasites per reaction. Each run also included one reaction with molecular grade water as a negative control. Each DNA sample was evaluated in triplicate. Samples with cycle threshold (Ct) >40 were considered negative.

**Table 2:** Primer & probe sequence of real time PCR.

Primer & Probe	Sequence	Neucleotide position
**Forward Primer**	5’- GCGACGTCCGTGGAAAGAA-3’	77–95
**Reverse Primer**	5‘-GGCGGGTACACATTAGCAGAA-3’	122–142
**Probe**	5’-CAACGCGTATTCCC-3’	108–121

### Analytical sensitivity, linearity & reproducibility of real time PCR

To establish the minimum number of parasites that could be detected by the assay, a serial dilution of DNA extracted from *in vitro* cultured promastigotes (*L*. *donovani* MHOM/IN/80/DD8) was made from 10 ng to 1 fg of parasite DNA corresponding to 100,000 to 0.01 parasites per reaction [31]. All dilutions were assayed in triplicate. For intra-assay validation, three replicates of seven 10-fold DNA concentrations, from 100,000 parasites to 0.1 parasite per reaction were assessed in a single run. Similar dilutions were prepared and two independent runs performed for inter assay validation. Variability of the assay is reported as the coefficient of variation (CV; shown as the ratio of mean to standard deviation (SD)). Products of real time PCR were subjected to gel electrophoresis in 1.5% agarose gel for assessment of analytical specificity.

### Clinical sensitivity, specificity & statistical analysis

The clinical sensitivity and specificity of the *Leishmania* TaqMan qPCR assay and nested PCR for detecting and identifying *Leishmania* parasites were calculated considering clinical confirmation with positive rK39 RDT as the “gold standard” for VL and PKDL cases. For RVL cases, positive identification of parasites in spleen aspirate by microscopy was considered as “gold standard”. Sensitivity and specificity with 95% CI were calculated using exact binomial methods for proportions. Cohen’s kappa coefficient (k) was determined for agreement testing between two PCR methods. The values of Cohen’s k coefficients were interpreted according to Landis and Koch: 1.00–0.81: excellent; 0.80–0.61: good; 0.60–0.41: moderate; 0.40–0.21: weak; and 0.20–0.00: negligible agreement [10]. The McNemar test was performed to evaluate the discordance between the PCR methods. Repeated-measures analysis of variance (ANOVA) was used to compare parasite loads among all groups. A *p*-value < 0.05 was considered to indicate statistical significant differences. The minimum parasite load in real time PCR corresponding to the cumulative sensitivity in Ln-PCR was determined using receiver operating characteristic (ROC) curves.

## Results

### Clinical parameter of study participants

Clinical and demographic parameters of study participants are illustrated in Table 3.

**Table 3:** Demographic parameters of study participants.

Group	Age Mean±SD	Children N (%)	Adult N (%)	Male N (%)	Splenomegaly N (%)	Duration of fever (Days) Mean±SD	ESR (Minutes) Mean±SD	Hepatomegaly N (%)	Blackening N (%)	Pancytopenia N (%)	Macular skin lesion N (%)	Papular skin lesion N (%)	Nodular skin lesion N (%)
VL	20.20±12.57	22 (55)	18 (45)	24 (60)	39 (97.5)	22.23±15.78	102.50±31.32	30 (75)	37 (92.5)	19 (47.5)	N/A	N/A	N/A
RVL	28.20±13.08	3 (30)	7 (70)	6 (60)	10 (100)	17.43±12.19	93.43±35.67	7 (70)	10 (100)	6 (60)	N/A	N/A	N/A
Cured VL	30.1±12.07	3 (15)	17(85)	9 (45)	N/A	N/A	N/A	N/A	N/A	N/A	N/A	N/A	N/A
PKDL	29.95±16.28	11 (27.5)	29 (72.5)	29 (72.5)	N/A	N/A	N/A	N/A	N/A	N/A	38 (95)	2 (5)	0 (0)
Cured PKDL	29.95±16.28	11 (27.5)	29 (72.5)	29 (72.5)	N/A	N/A	N/A	N/A	N/A	N/A	N/A	N/A	N/A
EC	31.60±8.71	0 (0)	80 (100)	50 (62.5)	N/A	N/A	N/A	N/A	N/A	N/A	N/A	N/A	N/A
TB	39.00±14.49	0 (0)	10 (100)	5 (50)	N/A	22.93±9.49	106.31±19.14	N/A	N/A	N/A	N/A	N/A	N/A

### Analytical sensitivity, linearity & reproducibility of the real time PCR

**Fig 1:**
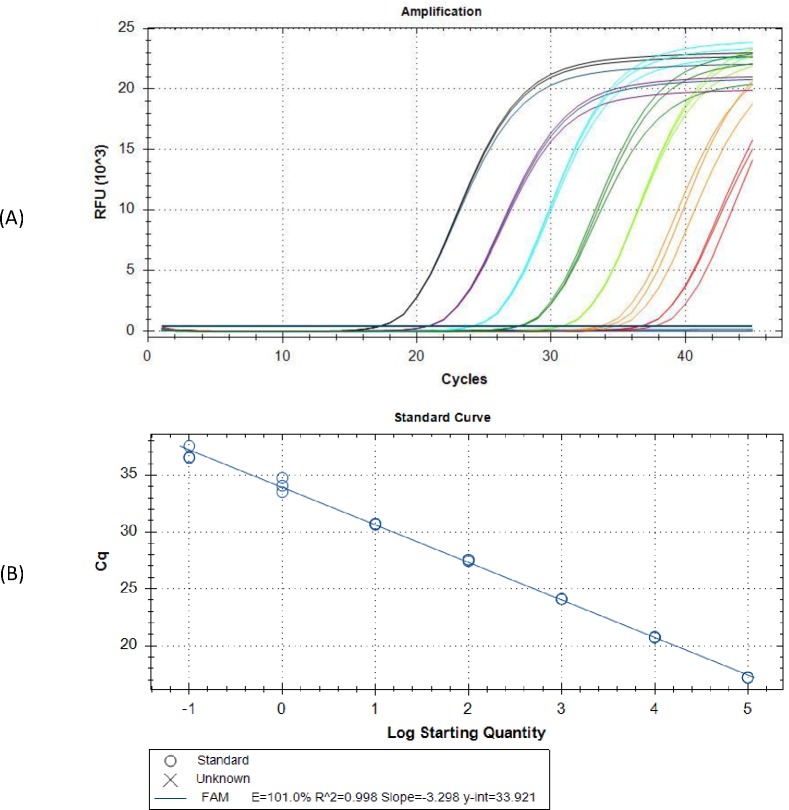
Technical performance and range of detection of the Leishmania real-time PCR assay. In **(A)** DNA was extracted from serial dilutions of cultured *L*. *donovani*, ranging from 1 x 10^5^ to 1x 10^−1^/ ml, and subjected to real time PCR. Amplification curves are shown for each sample, with each parasite concentration depicted by a differing color. In **(B)** the mean Ct values are plotted from triplicates tested against serial dilutions containing 10ng to 10fg of *L*.*donovani* genomic DNA per reaction. Each point represents the Ct of an individual sample, with the plot of Ct values and parasite equivalent fitting a linear function (**R**^2^ _0.998).

The novel real time PCR assay detected as low as 10 fg of *Leishmania donovani* genomic DNA per reaction, a quantity that corresponds to 0.1 parasite (Fig 1A and Table 3). The standard curve generated by serial dilution of parasite DNA was linear over 7 log range with correlation coefficient (R^2^) of 0.998 and absolute efficiency (Fig 1B). Also, nonappearance of any detectable Ct value in the non-template control signified complete absence of non-specific reactions or contamination Thus, the assay was highly specific for *Leishmania*. The intra assay CV of Ct values among the replicates were 0.06, 0.17, 0.00, 0.26, 0.13, 1.85, and 0.93% for seven different concentrations, respectively. Increased variation is observed with the lowest quantities of parasite DNA. Reproducibility of the assay was assessed as inter assay variation of Ct values for same dilution series in two independent runs. Inter assay coefficients of variations were found to be 3.63, 2.14, 1.88, 1.37, 1.71, 0.47 and 2.95% respectively, indicating high reproducibility (Table 4).

**Table 4:** Repeatability and reproducibility of the real time PCR assay.

	*Intra assay variation of Ct values*						*Inter assay variation of Ct values*				
Parasite Load	Replicate 1	Replicate 2	Replicate 3	Mean	SD	CV%	Assay 1	Assay 2	Mean	SD	CV%
**1x10 ^5^ **	17.20	17.21	17.19	17.20	0.01	0.06	17.20	18.10	17.64	0.64	3.63
**1x10 ^4^ **	20.79	20.72	20.74	20.75	0.04	0.17	20.75	21.39	21.07	0.45	2.14
**1x10 ^3^**	24.11	24.11	24.11	24.11	0.00	0.00	24.11	24.76	24.43	0.46	1.88
**1x10 ^2^**	27.56	27.43	27.55	27.51	0.07	0.26	27.51	28.04	27.78	0.38	1.37
**1x10 ^1^**	30.73	30.68	30.65	30.69	0.04	0.13	30.69	31.44	31.07	0.53	1.71
**1x10 ^0^**	34.77	33.51	34.26	34.18	0.63	1.85	34.18	34.41	34.30	0.16	0.47
**1x10 ^-1^ **	36.59	37.56	36.51	36.89	0.34	0.93	36.89	38.46	37.67	1.11	2.95

SD, standard deviation; CV, Co-efficient of variation

### Clinical sensitivity and specificity of real time PCR

**Fig 2:**
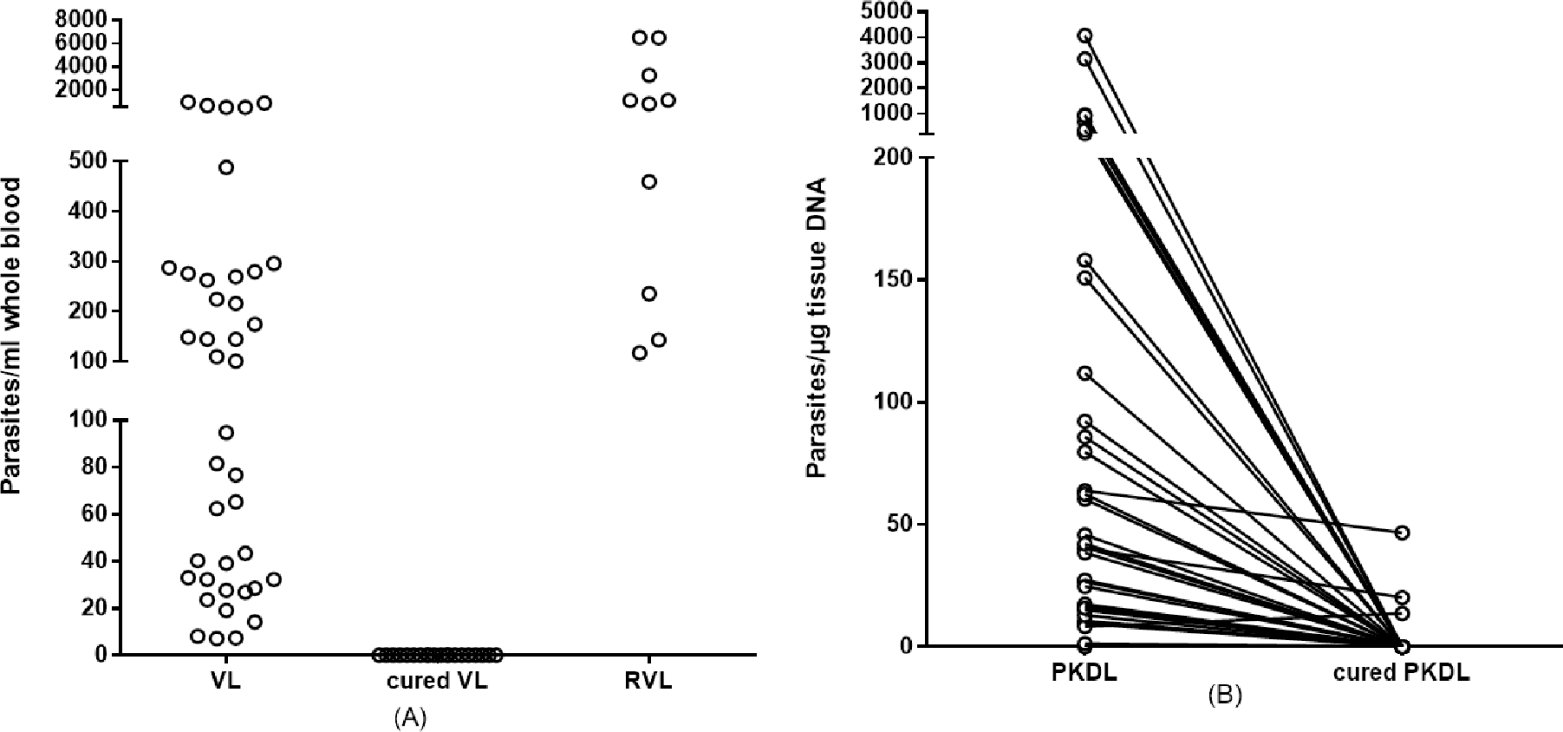
Parasite DNA can be detected in active, but not cured, disease states. In (A), parasite burden in blood samples from VL (n = 40), VL patients deemed to be cured by symptomatic response to treatment (n = 40) and RVL patients (n = 10) were measured. Each point indicates the data obtained from each individual sample. In (B), parasite burden in skin samples from PKDL patients before or after treatment (n = 40) were measured. Each point indicates the data obtained from each individual sample, and before and after data for each patient are linked by the connecting line.

The real time PCR assay successfully amplified parasite DNA in all 40 clinically confirmed VL samples, rendering 100% sensitivity (95% CI, 91.19–100%) (Fig 2A). Parasite DNA was not detected in any of the cured VL samples, indicating complete elimination as a result of effective treatment. Circulating parasite DNA was, however, detected in all 10 of the RVL samples evaluated, indicating 100% sensitivity for RVL (95% CI, 69.15–100%). The assay detected 34 of 40 PKDL samples with 85.00% sensitivity (95% CI, 70.16–94.29%) (Fig 2B). Thus, importantly, and consistent with parasitological reports of *L*. *donovani* persistence in the skin of PKDL patients, our real time PCR assay detected parasite DNA in the majority of skin samples from PKDL patients. Parasite DNA could be detected in 7.5% (3 of 40) of the cured PKDL samples. Product was not amplified in any of the endemic control or TB samples by the PCR assay, such that cross reactivity with *M*. *tuberculosis* was not observed and a specificity of 100% was indicated (95% CI, 95.98–100).

### Clinical sensitivity and specificity of *Leishmania* nested PCR

The *Leishmania* nested PCR detected parasite DNA in 31 out of 40 VL samples and in all 10 RVL samples (Table 5). Therefore, nested PCR achieved 77.50% sensitivity for VL (95% CI, 61.55–89.16%) and 100% sensitivity for RVL (95%CI, 69.15–100%). The nested PCR detected parasite DNA in only 21 of 40 PKDL samples (52.50% sensitivity (95% CI, 36.13–68.49%)). Elimination of parasite DNA to levels below the limit of detection was found in all of the cured VL samples and 97.5% (39 of 40) of the cured PKDL samples. The nested PCR assay was 100% (95% CI, 95.98–100) specific and amplified product in none of the endemic control samples. Similarly, it did not show cross reactivity with any of the TB samples.

**Table 5:** Sensitivity and specificity of Ln-PCR and real time PCR in detecting *L*. *donovani* in clinical samples.

*Assay*	*Sensitivity n (%)*, *95%CI*				*Specificity n (%)*, *95%CI*		
	VL N = 40	RVL N = 10	PKDL N = 40	All groups (VL+PKDL+RVL) N = 90	Endemic Control N = 80	TB N = 10	All groups (Endemic Control+TB) N = 90
**Real Time PCR**	40 (100), (91.19–100)	10 (100), (69.15–100)	34 (85), (70.16–94.29)	84 (93.33), (86.05–97.51)	0 (100), (95.98–100)	0 (100), (95.98–100)	90 (100), (95.98–100)
**Ln-PCR**	31 (77.50), (61.55–89.16)	10 (100), (69.15–100)	21 (52.50), 36.13–68.49)	62 (68.89), (58.26–78.23)	0 (100), (95.98–100)	0 (100), (95.98–100)	90 (100), (95.98–100)

### Comparison between real time and nested PCR

**Fig 3:**
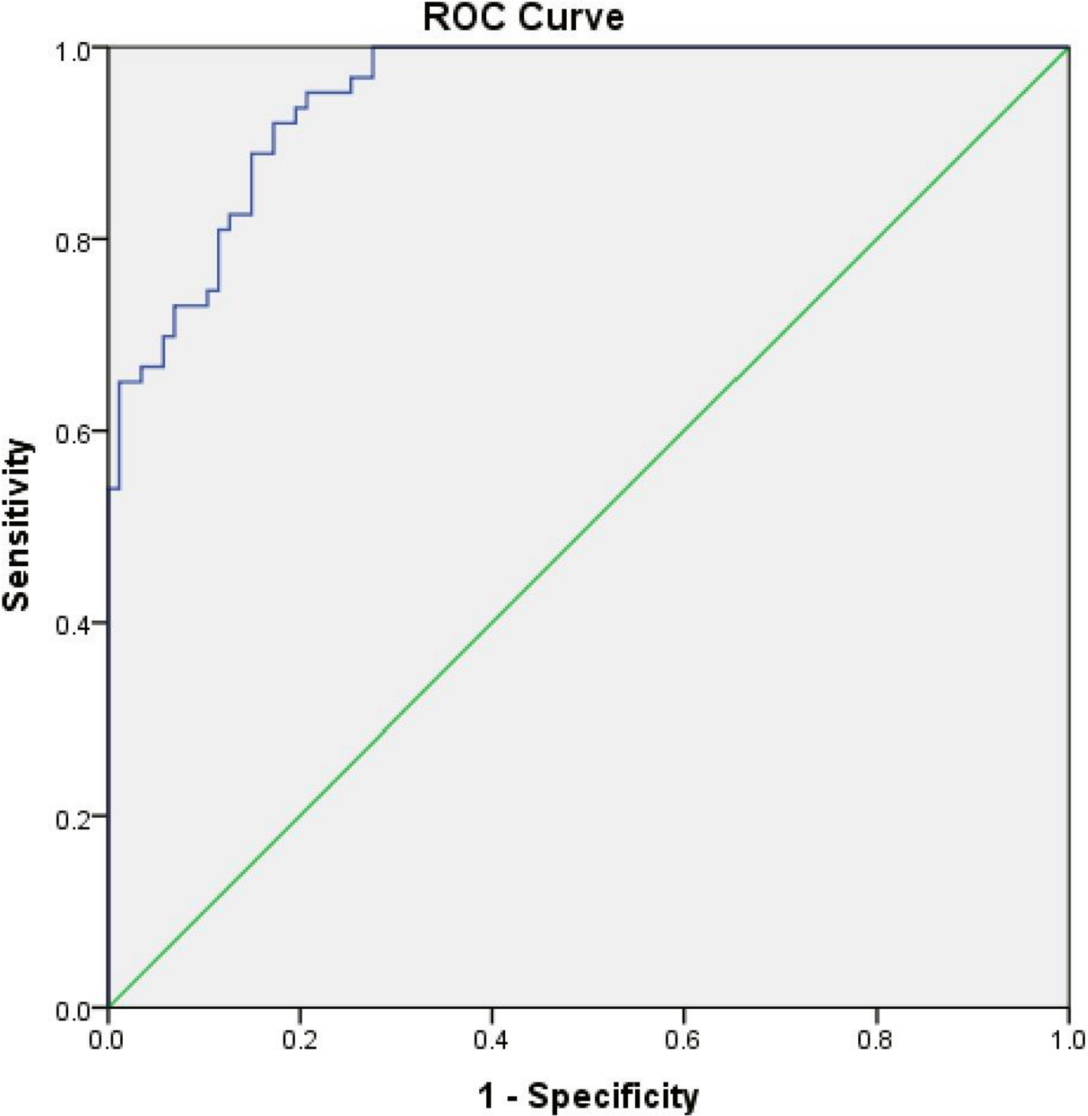
Receiver operating characteristic (ROC) curve for Ln-PCR for the detection of *Leishmania* parasites in clinical samples.

The cumulative sensitivities of the real time PCR and Ln-PCR assay were 93.33% (95% CI, 86.05–97.51%) and 68.89% (95% CI, 58.26–78.23%), respectively. Cumulative specificity of both assays was 100% (95% CI, 95.98–100%). Comparisons of sensitivity and specificities of Ln-PCR and real time PCR are illustrated in Table 4. While, with a kappa value of 0.273, a weak agreement was indicated between the assays, the McNemar’s test indicated significant discordance between the real time PCR and Ln-PCR in the detection of *L*. *donovani* in clinical samples (χ2 = 20.05; P<0.0001). ROC analysis indicated a much weaker ability for the nested PCR assay to detect *L*. *donovani*, with a minimum DNA equivalent to 64.6 parasites (as quantitated by real time PCR) being detected by Ln-PCR (Fig 3). These comparisons indicate enhanced performance of the real time PCR assay over nested PCR. Area under the curve (AUC) for PSA is 0.949 with p = 0.00.

### Quantification of parasites in blood and skin tissue by real time PCR

The real time PCR detected a range of parasites from 7.11 parasites/mL up to 962.22 parasites/mL in VL samples, with mean of 192.69 parasites/mL blood (Fig 2A). DNA quantities in RVL samples were higher, ranging from 117.11 parasites/mL blood up to 6522.0 parasites/mL blood and with a mean of 2028.24 parasites/mL blood. In contrast, parasites were at a minimum below 0.1 parasites, 10fg DNA/ml detection level in all cured VL patients. Thus, although these samples were not matched, our data indicate that while parasites are readily detected in the circulation of VL patients they cleared by effective treatment. In contrast, parasite levels appear to become even more elevated in RVL cases.

Among 40 PKDL patients, 95% (38/40) were presenting clinically with hypo-pigmented macules and papular lesions were found in 5% (2/40) patients. High variability in parasite burden is indicated by DNA extracted from skin biopsies of PKDL patients, with the real time PCR assay detecting as low as 1.38 parasites/ μg tissue DNA and as high as 4065.89 parasites/ μg tissue DNA, with a mean of 295.46 parasites/ μg tissue DNA (Fig 2B). Following treatment, samples from only 3 patients remained positive and parasites could not be detected in the vast majority of PKDL patients. Taken together, our data indicate the utility of the real time PCR assay in monitoring treatment and parasitological cure of both VL and PKDL patients.

## Discussion

Over the last decade a variety of real time PCR assays have been developed in the search for appropriate tools for detection of different *Leishmania* species. The efficacy of those assays has varied, however, depending on the copy number of target sequence and the specimen type [32]. In this study, we evaluated the diagnostic accuracy of a novel real time PCR across multiple presentations associated with *L*. *donovani* infection. While parasite DNA was readily detected at extremely low levels in the blood of VL patients and the skin of PKDL patients, treatment cleared these burdens. Importantly, parasites re-emerged in relapsed VL patients. Together, our data support the use of this sensitive and specific real time PCR assay to detect and monitor *L*. *donovani* infection in both VL and PKDL patients.

By spiking *Leishmania* promastigote DNA directly into reactions the analytical sensitivity of the real time PCR assay was demonstrated corresponding to 0.1 parasite per reaction. The lower detection limit is slightly different from that reported in our original study, where the sensitivity reached 0.01 parasite per mL promastigote spiked blood [11], a variance likely arising due to small modifications that commonly occur at the lower range of any standard curve. Regardless, the real time PCR assay remains extremely sensitive in the detection of circulating parasite DNA, detecting at much lower levels than Ln-PCR. The clinical sensitivity of the assay was assessed in samples from three distinct group of patients (VL, PKDL and RVL). The assay showed 100% (95% CI) sensitivity in detecting parasite DNA in VL and RVL samples, which is promising when is compared to former qPCR assays carried out with peripheral blood. In a SYBR green reaction based assay with a commonly used kDNA RV1/RV2 primer system, 91.3% sensitivity and 29.6% specificity was reported [26]. A different SYBR green based reaction demonstrated only 79% sensitivity targeting another kDNA minicircle sequence [21], rising to 94.7% sensitivity when targeting a genomic single copy polymerase gene by Taqman chemistry [33]. 95.7% sensitivity and 100% sensitivity are also reported with parasitologically confirmed patients in two recent SYBR green reaction based studies targeting kinetoplast DNA [34,35].

In contrast, the SSU rDNA targeted Ln-PCR has shown sensitivities ranging between 79–97% [25][36][37]. The use of DNA extracted from the buffy coat, rather than from whole blood, might have contributed to the improved sensitivity observed in our study. In accordance with previous reports, sensitivity achieved by the Ln-PCR in VL samples was 77.50% (95% CI) and in RVL samples the sensitivity was 100% (95%CI). Moreover, the ROC analysis indicated that a minimum of 64.6 parasite need to be present in clinical samples to reach the maximum sensitivity and specificity by Ln-PCR, which further suggests the supremacy of real time PCR in diagnosis of VL and PKDL.

In detecting *Leishmania* parasites in skin biopsies from PKDL patients, the real time PCR achieved a sensitivity of 85.0% (95% CI), markedly higher than that observed with the Ln-PCR (52.50%). While the real time PCR assay appeared better, it is worth noting that the sensitivity of our SSU-rDNA targeted Ln PCR was, however, lower than other reported nested PCR assays. For example, a kDNA directed nested PCR with skin biopsy showed 94.5% and 93% sensitivities in two different studies [17][23], while another nested PCR assay targeting ribosomal ITS region achieved 91.9% sensitivity with skin biopsy specimens, albeit providing the lowest sensitivity with macular lesions [38].

Despite research into VL, to date, very few real time PCR assays have been evaluated for their ability to diagnosis of PKDL. In a SYBR green reaction based real time PCR targeting kDNA minicircle, absolute sensitivity was achieved with skin punch biopsies with the mean parasite load 21,855 parasites/μg tissue DNA[16]. A higher accuracy for the detection and quantification of parasites in skin biopsies from PKDL patients compared to slit smear microscopy was reported with a kDNA targeted SYBR Green reaction based real time PCR assay where the mean parasite load was 9,502 parasites/μg tissue DNA [31]. In both of these studies, parasite rich nodular/papular lesions were the prevalent presentation whereas in our study 95% of the lesions appeared clinically as macular, the manifestation that is claimed to carry the least number of parasites [16]. As etiopathogenesis of PKDL is still obscure and the causes of this parasite load variation with different clinical manifestation is yet to be revealed. However, few studies associated the lower parasitic load in macular PKDL with strong CMI and low antibody level [39]. We therefore speculate that the lower parasitic load and sensitivity of real time PCR in our study is associated with prevalence of macular lesions. This also depicts the predominance of hypo-pigmented macules of Bangladeshi PKDL patients in agreement with previous reports [40].

According to the national guideline of Bangladesh, new VL cases are diagnosed by the recognition of clinical signs and symptoms in conjunction with a positive result in rK39 RDT. Splenic aspirate, to generate material for microscopy, is not recommended because of the risk of fatal hemorrhage. rK39 RDT are incompatible for the confirmation of reactivated disease because they remain positive in cured VL patients, however, and therefore, despite the risks, spleen aspirations are currently obligatory for the diagnosis of RVL [41]. There is also currently no standard reference test for the diagnosis of PKDL, which is diagnosed by the combination of clinical signs/symptoms, positive rK39 RDT, and accounting for a history of VL and visits to VL-endemic areas [14]. The treatment modality of VL and PKDL is chemotherapeutics dependent. In Bangladesh, VL patients are currently treated by single dose liposomal amphotericin B and PKDL patients are treated by miltefosine, 100 mg/day, orally for 12 weeks for patients weighing > 25 kg and 50 mg orally per day for 12 weeks for patients weighing < 25 kg [9]. As chemotherapeutic treatments of VL, RVL and PKDL are all vigorous and expensive [42], thorough clinical observation and an accurate monitoring test could be used to optimize the therapies. The real time PCR assay described here appears suited for this purpose as it detected parasites with absolute specificity in peripheral blood and skin biopsies, respectively, while offering a minimally invasive and confirmatory method. It also overcomes some of the practical limitations of nested PCR as it eliminates both the need for a secondary step and visualization of product by gel electrophoresis after reactions are complete. These reduce the risk of contamination and reduce the time required for obtaining results, allowing more samples to be analyzed in both a single assay run and within a set period of time. Among the current limitations of the real time PCR assay that could limit integration into a routine diagnostic algorithm is the requirement for an established referral laboratory infra-structure [43]. As it offers diverse range of application and operational advantages, however, the interest in real time PCR is increasing and infrastructure development costs could eventually be divided across several disease fields. Indeed, in Bangladesh real time PCR facilities have already reached the district level.

Apart from its suitability in disease diagnosis, real time PCR can also be advantageous in assessing response to therapy [18]. A study in India demonstrated a significant correlation between parasite load and clinical outcome in patients undergoing treatment with L-AmpB [43]. In another study it is indicated that the persistence of more than 1 parasite/mL blood after VL treatment is associated with a high risk of relapse[44] and several studies show an increase above 10 parasites/mL of blood after treatment is indicative of disease relapse[43][45][46]. The real time PCR was also proficient in monitoring parasite kinetics, as continuing remission of the infection could be deduced in cured PKDL cases. Differences in parasite load between VL and RVL groups was significant and is consistent with a previous study [43], an observation that suggests future investigation to establish parasite load as a prognostic marker for disease relapse is warranted.

The ability of the real time PCR assay to quantify parasite genomes could also potentially allow use in monitoring infection dynamics, irrespective of disease state. As such, the real time PCR is also convenient in the detection of very low parasitic loads typically observed in asymptomatic carriage of parasites [11][44]. An epidemiological study in India demonstrated that asymptomatic individuals with high and persistent parasite burdens are more likely to progress to active disease [30] and Mary *et al* implied that in Mediterranean basin the threshold between asymptomatic to symptomatic state falls within the range of 1–8 parasite per mL of blood [44]. These parasite levels are readily within the linear discriminatory range of the standard curve associated with our real time PCR assay. Also, in our study VL and PKDL showed similar parasite burdens, suggesting that they might play equal roles in parasite transmission.

The real time PCR assay in this study was expedient in detection and quantification of considerably low parasite loads in samples from VL, RVL and PKDL patients. Further evaluation with expanded numbers of samples from individuals with different clinical manifestations should be considered, with a prospective study in which individuals would transition between the disease states appearing particularly beneficial. In conclusion, the real time PCR assay we described could be readily implemented to alter the routine laboratory diagnosis paradigm of VL and its related complications. This has the potential to be a significant part of programs to eliminate Kala-azar from affected countries.

## Supporting information

S1 Table Result of Ln-PCR and Real time PCR in buffycoat DNA of VL patients. (DOCX)

S2 Table Result of Ln-PCR and Real time PCR in buffycoat DNA of RVL patients. (DOCX)

S3 Table Result of Ln-PCR and Real time PCR in buffycoat DNA of cured VL patients. (DOCX)

S4 Table Clinical presentation and comparative result of Ln-PCR and Real time PCR in DNA from skin biopsies of PKDL and cured PKDL patients. (DOCX)

S5 Table Result of Ln-PCR and Real time PCR in buffycoat DNA of endemic controls. (DOCX)

S6 Table Result of Ln-PCR and Real time PCR in buffycoat DNA of TB patients. (DOCX)
