# Supplementary material for: Real-time PCR in detection and quantitation of Leishmania donovani for the diagnosis of Visceral Leishmaniasis patients and the monitoring of their response to treatment
Source: PLoS One. 2017 Sep 28;12(9):e0185606. doi: 10.1371/journal.pone.0185606 (PMC5619796; doi:10.1371/journal.pone.0185606)
Supplement: S1 Table — (DOCX) [file pone.0185606.s001.docx]

**Supporting information**

**S1 Table: Result of Ln-PCR and Real time PCR in buffycoat DNA of VL patients.**

| SL | Age | Sex | DNA concentration (ng/µL) | Ln-PCR | Real time PCR | |
| --- | --- | --- | --- | --- | --- | --- |
|  |  |  |  |  | **Ct** | **Parasites/mL Whole Blood** |
| 1 | 40 | M | 1.8 | Positive | 36.99 | 94.78 |
| 2 | 28 | M | 4.4 | Positive | 33.16 | 513.78 |
| 3 | 30 | M | 5.7 | Negative | 36.99 | 93.36 |
| 4 | 15 | M | 12.9 | Positive | 34.44 | 224.44 |
| 5 | 10 | M | 3.8 | Positive | 34.20 | 262.44 |
| 6 | 11 | F | 1.9 | Positive | 34.12 | 276.00 |
| 7 | 9 | M | 1.4 | Positive | 37.10 | 40.24 |
| 8 | 25 | M | 10.8 | Positive | 32.35 | 864.22 |
| 9 | 6 | F | 8.2 | Positive | 34.15 | 269.78 |
| 10 | 45 | M | 13.2 | Positive | 32.19 | 962.22 |
| 11 | 35 | F | 1.5 | Positive | 35.55 | 109.73 |
| 12 | 7 | F | 9.7 | Negative | 37.63 | 28.53 |
| 13 | 12 | F | 13.8 | Positive | 34.50 | 216.00 |
| 14 | 28 | M | 6.3 | Positive | 38.24 | 19.11 |
| 15 | 8 | M | 11.2 | Positive | 35.08 | 148.40 |
| 16 | 9 | F | 4.6 | Positive | 39.76 | 7.11 |
| 17 | 25 | M | 16.5 | Positive | 37.14 | 39.18 |
| 18 | 22 | M | 4.2 | Negative | 39.53 | 8.22 |
| 19 | 3.5 | F | 2.8 | Positive | 38.69 | 14.22 |
| 20 | 2.5 | F | 5.9 | Negative | 39.71 | 7.33 |
| 21 | 3 | F | 2.4 | Positive | 35.68 | 100.73 |
| 22 | 48 | M | 7.2 | Positive | 37.44 | 32.31 |
| 23 | 7 | M | 2.8 | Negative | 37.41 | 33.02 |
| 24 | 28 | M | 83 | Negative | 31.60 | 76.80 |
| 25 | 40 | M | 5.5 | Positive | 36.35 | 65.29 |
| 26 | 38 | M | 10.9 | Positive | 35.12 | 144.56 |
| 27 | 9 | F | 4.1 | Positive | 34.01 | 296.00 |
| 28 | 12 | F | 23.3 | Positive | 34.83 | 174.13 |
| 29 | 15 | F | 8 | Positive | 34.06 | 287.33 |
| 30 | 35 | F | 3.5 | Positive | 37.73 | 26.87 |
| 31 | 8 | F | 4 | Negative | 38.44 | 23.56 |
| 32 | 17 | M | 3.4 | Positive | 32.75 | 666.89 |
| 33 | 19 | F | 2.9 | Positive | 33.24 | 488.22 |
| 34 | 10 | M | 3.4 | Positive | 36.42 | 62.44 |
| 35 | 29 | M | 1.6 | Positive | 33.16 | 514.22 |
| 36 | 3.5 | F | 3.3 | Positive | 34.10 | 279.78 |
| 37 | 14 | M | 2.8 | Positive | 37.68 | 27.67 |
| 38 | 12 | M | 2.6 | Negative | 37.44 | 32.29 |
| 39 | 25 | M | 4.4 | Negative | 35.12 | 144.67 |
| 40 | 30 | M | 3.1 | Positive | 36.01 | 81.56 |
