# Supplementary material for: Real-time PCR in detection and quantitation of Leishmania donovani for the diagnosis of Visceral Leishmaniasis patients and the monitoring of their response to treatment
Source: PLoS One. 2017 Sep 28;12(9):e0185606. doi: 10.1371/journal.pone.0185606 (PMC5619796; doi:10.1371/journal.pone.0185606)
Supplement: S2 Table — (DOCX) [file pone.0185606.s002.docx]

**Supporting information**

**S2 Table: Result of Ln-PCR and Real time PCR in buffycoat DNA of RVL patients.**

| SL | Age | Sex | DNA concentration (ng/µL) | Ln-PCR | Real time PCR | |
| --- | --- | --- | --- | --- | --- | --- |
|  |  |  |  |  | **Ct** | **Parasites/mL Whole Blood** |
| 1 | 18 | F | 124.4 | Positive | 34.61 | 117.11 |
| 2 | 58 | F | 84.5 | Positive | 31.96 | 459.56 |
| 3 | 30 | M | 122.9 | Positive | 28.88 | 3253.33 |
| 4 | 17 | M | 82.5 | Positive | 31.08 | 802.44 |
| 5 | 32 | F | 120.8 | Positive | 30.57 | 1109.78 |
| 6 | 15 | F | 95.8 | Positive | 27.79 | 6495.56 |
| 7 | 27 | M | 87.1 | Positive | 30.54 | 1121.78 |
| 8 | 16 | M | 106.1 | Positive | 27.79 | 6522.00 |
| 9 | 35 | M | 168.6 | Positive | 33.79 | 143.11 |
| 10 | 34 | M | 86.7 | Positive | 33.90 | 235.56 |
