# Supplementary material for: Real-time PCR in detection and quantitation of Leishmania donovani for the diagnosis of Visceral Leishmaniasis patients and the monitoring of their response to treatment
Source: PLoS One. 2017 Sep 28;12(9):e0185606. doi: 10.1371/journal.pone.0185606 (PMC5619796; doi:10.1371/journal.pone.0185606)
Supplement: S3 Table — (DOCX) [file pone.0185606.s003.docx]

**Supporting information**

**S3 Table: Result of Ln-PCR and Real time PCR in buffycoat DNA of cured VL patients.**

| SL | Age | Sex | Duration after treatment | DNA concentration (ng/µL) | Ln-PCR | Real time PCR | |
| --- | --- | --- | --- | --- | --- | --- | --- |
|  |  |  |  |  |  | **Ct** | **Parasites/mL Whole Blood** |
| 1 | 50 | Male | 1 Year | 51.5 | Negative | ND | N/A |
| 2 | 46 | Female | 1 year | 40.8 | Negative | ND | N/A |
| 3 | 25 | Female | 1 year | 26.8 | Negative | ND | N/A |
| 4 | 35 | Male | 1 year | 26.7 | Negative | ND | N/A |
| 5 | 32 | Female | 1 year | 33 | Negative | ND | N/A |
| 6 | 36 | Female | 1 year | 58.2 | Negative | ND | N/A |
| 7 | 20 | Female | 1 year | 51.4 | Negative | ND | N/A |
| 8 | 35 | Male | 1 year | 71.6 | Negative | ND | N/A |
| 9 | 34 | Male | 1 year | 52.3 | Negative | ND | N/A |
| 10 | 20 | Male | 1 year | 55.8 | Negative | ND | N/A |
| 11 | 18 | Female | 2 months | 50.1 | Negative | ND | N/A |
| 12 | 58 | Female | 2 months | 20 | Negative | ND | N/A |
| 13 | 17 | Male | 2 months | 27.7 | Negative | ND | N/A |
| 14 | 32 | Female | 2 months | 65.2 | Negative | ND | N/A |
| 15 | 15 | Female | 2 months | 28.3 | Negative | ND | N/A |
| 16 | 27 | Male | 2 months | 56.7 | Negative | ND | N/A |
| 17 | 16 | Male | 2 months | 19.2 | Negative | ND | N/A |
| 18 | 18 | Female | 2 months | 77.8 | Negative | ND | N/A |
| 19 | 28 | Female | 2 months | 46.1 | Negative | ND | N/A |
| 20 | 40 | Male | 2 months | 33.8 | Negative | ND | N/A |

*ND=Not detected; N/A=Not applicable*
