# Supplementary material for: Real-time PCR in detection and quantitation of Leishmania donovani for the diagnosis of Visceral Leishmaniasis patients and the monitoring of their response to treatment
Source: PLoS One. 2017 Sep 28;12(9):e0185606. doi: 10.1371/journal.pone.0185606 (PMC5619796; doi:10.1371/journal.pone.0185606)
Supplement: S4 Table — (DOCX) [file pone.0185606.s004.docx]

**Supporting information**

**S4 Table: Clinical presentation and comparative result of Ln-PCR and Real time PCR in DNA from skin**

| SL | Age | Sex | Clinical Presentation | Duration after treatment | DNA concentration (ng/µL) | | | Ln-PCR | | Real time PCR | | | |
| --- | --- | --- | --- | --- | --- | --- | --- | --- | --- | --- | --- | --- | --- |
|  |  |  |  |  |  |  |  |  |  | PKDL | | Cured PKDL | |
|  |  |  |  |  | PKDL | | Cured PKDL | PKDL | Cured PKDL | Ct | Parasites/µg tissue DNA | Ct | Parasites/µg tissue DNA |
| 1 | 50 | M | Papular | 2 months | 3.4 | 4.5 | | Positive | Negative | 28.42 | 3147.71 | ND | N/A |
| 2 | 56 | F | Macular | 2 months | 7 | 5.8 | | Negative | Negative | ND | N/A | ND | N/A |
| 3 | 35 | M | Macular | 2 months | 3.4 | 10.2 | | Positive | Negative | 31.1 | 682.97 | ND | N/A |
| 4 | 28 | M | Macular | 2 months | 6.2 | 15.8 | | Positive | Negative | 32.07 | 215.25 | ND | N/A |
| 5 | 32 | M | Macular | 2 months | 10.5 | 6.2 | | Positive | Negative | 34.08 | 40.31 | ND | N/A |
| 6 | 36 | M | Macular | 2 months | 9 | 1.2 | | Positive | Positive | 31.41 | 215.81 | 38.39 | 20.20 |
| 7 | 20 | M | Macular | 2 months | 6.6 | 10.3 | | Positive | Negative | 34.18 | 60.49 | ND | N/A |
| 8 | 45 | M | Macular | 2 months | 5.9 | 6.6 | | Positive | Negative | 35.81 | 26.71 | ND | N/A |
| 9 | 18 | M | Macular | 2 months | 3.4 | 9.6 | | Negative | Negative | ND | N/A | ND | N/A |
| 10 | 55 | F | Macular | 2 months | 3.3 | 9.3 | | Negative | Negative | ND | N/A | ND | N/A |
| 11 | 55 | M | Papular | 2 months | 5.7 | 29.9 | | Positive | Negative | 27.07 | 4065.89 | ND | N/A |
| 12 | 42 | F | Macular | 2 months | 5 | 9.1 | | Positive | Negative | 33.59 | 111.97 | ND | N/A |
| 13 | 38 | M | Macular | 2 months | 1.7 | 22.9 | | Positive | Negative | 34.95 | 150.91 | ND | N/A |
| 14 | 19 | M | Macular | 2 months | 4.1 | 15.2 | | Negative | Negative | ND | N/A | ND | N/A |
| 15 | 18 | M | Macular | 2 months | 1.4 | 8.3 | | Negative | Negative | 38.01 | 10.63 | ND | N/A |
| 16 | 55 | M | Macular | 2 months | 4.3 | 6.6 | | Negative | Negative | 38.15 | 9.60 | ND | N/A |
| 17 | 60 | M | Macular | 2 months | 3.6 | 11.6 | | Positive | Negative | 33.56 | 158.21 | ND | N/A |
| 18 | 35 | F | Macular | 2 months | 4.7 | 7.6 | | Positive | Negative | 36.17 | 27.28 | ND | N/A |
| 19 | 42 | M | Macular | 2 months | 3.9 | 10 | | Negative | Negative | 36.67 | 24.69 | ND | N/A |
| 20 | 22 | F | Macular | 2 months | 5.3 | 6.3 | | Negative | Negative | ND | N/A | ND | N/A |
| 21 | 30 | F | Macular | 2 months | 3.4 | 10.7 | | Positive | Negative | 35.24 | 63.91 | ND | N/A |
| 22 | 23 | M | Macular | 2 months | 4.5 | 0.84 | | Positive | Negative | 31.74 | 356.86 | 37.98 | 46.66 |
| 23 | 30 | M | Macular | 2 months | 8.6 | 7.4 | | Positive | Negative | 37.18 | 8.38 | ND | N/A |
| 24 | 18 | F | Macular | 2 months | 6.8 | 0.47 | | Negative | Negative | 34.81 | 40.86 | 39.56 | 13.74 |
| 25 | 32 | F | Macular | 2 months | 2.3 | 23.7 | | Negative | Negative | 35.41 | 85.93 | ND | N/A |
| 26 | 55 | M | Macular | 2 months | 4.2 | 21.5 | | Negative | Negative | ND | N/A | ND | N/A |
| 27 | 14 | M | Macular | 2 months | 5 | 10.3 | | Negative | Negative | 33.12 | 92.22 | ND | N/A |
| 28 | 11 | F | Macular | 2 months | 2.3 | 10.8 | | Negative | Negative | 36.72 | 16.46 | ND | N/A |
| 29 | 13 | M | Macular | 2 months | 3.9 | 5.3 | | Positive | Negative | 39.48 | 1.38 | ND | N/A |
| 30 | 13 | M | Macular | 2 months | 0.8 | 6.4 | | Positive | Negative | 37.11 | 38.47 | ND | N/A |
| 31 | 13 | M | Macular | 2 months | 4.9 | 6.6 | | Negative | Negative | 36.88 | 17.43 | ND | N/A |
| 32 | 58 | M | Macular | 2 months | 1.7 | 8.9 | | Negative | Negative | 35.66 | 79.74 | ND | N/A |
| 33 | 11 | M | Macular | 2 months | 1.9 | 10.9 | | Negative | Negative | 37.11 | 15.13 | ND | N/A |
| 34 | 12 | F | Macular | 2 months | 0.8 | 8.9 | | Negative | Negative | 36.76 | 45.81 | ND | N/A |
| 35 | 37 | M | Macular | 2 months | 4.1 | 18.9 | | Positive | Negative | 30.19 | 951.80 | ND | N/A |
| 36 | 10 | M | Macular | 2 months | 1 | 5.7 | | Positive | Negative | 36.55 | 42.20 | ND | N/A |
| 37 | 12 | F | Macular | 2 months | 3.9 | 16.2 | | Negative | Negative | 36.31 | 12.90 | ND | N/A |
| 38 | 13 | M | Macular | 2 months | 0.4 | 7.6 | | Negative | Negative | 34.90 | 62.50 | ND | N/A |
| 39 | 18 | M | Macular | 2 months | 3.4 | 5 | | Positive | Negative | 36.22 | 15.81 | ND | N/A |
| 40 | 14 | M | Macular | 2 months | 1 | 8.5 | | Positive | Negative | 32.15 | 922.10 | ND | N/A |

**biopsies of PKDL and cured PKDL patients.**

*ND=Not detected; N/A=Not applicable*
