# Supplementary material for: Real-time PCR in detection and quantitation of Leishmania donovani for the diagnosis of Visceral Leishmaniasis patients and the monitoring of their response to treatment
Source: PLoS One. 2017 Sep 28;12(9):e0185606. doi: 10.1371/journal.pone.0185606 (PMC5619796; doi:10.1371/journal.pone.0185606)
Supplement: S5 Table — (DOCX) [file pone.0185606.s005.docx]

**Supporting information**

**S5 Table: Result of Ln-PCR and Real time PCR in buffycoat DNA of endemic controls**

| SL | Age | Sex | DNA concentration (ng/µL) | Ln-PCR | Real Time PCR | |
| --- | --- | --- | --- | --- | --- | --- |
|  |  |  |  |  | Ct | Parasite Load |
| 1 | 22 | M | 88.8 | Negative | ND | NA |
| 2 | 32 | F | 88 | Negative | ND | NA |
| 3 | 42 | F | 33.4 | Negative | ND | NA |
| 4 | 23 | M | 18.8 | Negative | ND | NA |
| 5 | 31 | F | 21.3 | Negative | ND | NA |
| 6 | 31 | M | 126.7 | Negative | ND | NA |
| 7 | 24 | M | 44.9 | Negative | ND | NA |
| 8 | 41 | M | 38.7 | Negative | ND | NA |
| 9 | 24 | F | 35.2 | Negative | ND | NA |
| 10 | 23 | M | 37.3 | Negative | ND | NA |
| 11 | 43 | M | 109.9 | Negative | ND | NA |
| 12 | 33 | F | 82.8 | Negative | ND | NA |
| 13 | 41 | M | 71.5 | Negative | ND | NA |
| 14 | 40 | F | 44.9 | Negative | ND | NA |
| 15 | 18 | F | 69.5 | Negative | ND | NA |
| 16 | 37 | F | 46.6 | Negative | ND | NA |
| 17 | 21 | M | 34.2 | Negative | ND | NA |
| 18 | 34 | M | 24.2 | Negative | ND | NA |
| 19 | 28 | M | 59 | Negative | ND | NA |
| 20 | 46 | M | 132.1 | Negative | ND | NA |
| 21 | 20 | F | 32.2 | Negative | ND | NA |
| 22 | 32 | M | 116.2 | Negative | ND | NA |
| 23 | 43 | F | 99.5 | Negative | ND | NA |
| 24 | 23 | M | 63.9 | Negative | ND | NA |
| 25 | 32 | M | 68.5 | Negative | ND | NA |
| 26 | 31 | F | 64 | Negative | ND | NA |
| 27 | 25 | M | 42 | Negative | ND | NA |
| 28 | 41 | F | 26.4 | Negative | ND | NA |
| 29 | 23 | M | 50.9 | Negative | ND | NA |
| 30 | 22 | F | 44.8 | Negative | ND | NA |
| 31 | 43 | M | 51 | Negative | ND | NA |
| 32 | 31 | F | 57.2 | Negative | ND | NA |
| 33 | 40 | M | 75.6 | Negative | ND | NA |
| 34 | 42 | F | 78.2 | Negative | ND | NA |
| 35 | 18 | M | 56.4 | Negative | ND | NA |
| 36 | 36 | F | 65.4 | Negative | ND | NA |
| 37 | 19 | M | 59.7 | Negative | ND | NA |
| 38 | 33 | M | 56.1 | Negative | ND | NA |
| 39 | 27 | M | 57.3 | Negative | ND | NA |
| 40 | 46 | M | 49.6 | Negative | ND | NA |
| 41 | 21 | M | 60.2 | Negative | ND | NA |
| 42 | 32 | M | 86.6 | Negative | ND | NA |
| 43 | 43 | F | 33.7 | Negative | ND | NA |
| 44 | 24 | M | 48.6 | Negative | ND | NA |
| 45 | 32 | F | 65.1 | Negative | ND | NA |
| 46 | 30 | M | 66.9 | Negative | ND | NA |
| 47 | 25 | M | 33.1 | Negative | ND | NA |
| 48 | 40 | M | 43.5 | Negative | ND | NA |
| 49 | 24 | M | 41.6 | Negative | ND | NA |
| 50 | 23 | M | 53.7 | Negative | ND | NA |
| 51 | 44 | M | 44.1 | Negative | ND | NA |
| 52 | 32 | F | 53.5 | Negative | ND | NA |
| 53 | 40 | F | 53.3 | Negative | ND | NA |
| 54 | 42 | F | 33.4 | Negative | ND | NA |
| 55 | 18 | F | 53.7 | Negative | ND | NA |
| 56 | 36 | F | 83.4 | Negative | ND | NA |
| 57 | 19 | M | 40.9 | Negative | ND | NA |
| 58 | 35 | M | 44.9 | Negative | ND | NA |
| 59 | 28 | M | 47.3 | Negative | ND | NA |
| 60 | 47 | M | 41.4 | Negative | ND | NA |
| 61 | 22 | M | 49.1 | Negative | ND | NA |
| 62 | 31 | F | 22.5 | Negative | ND | NA |
| 63 | 43 | F | 39.6 | Negative | ND | NA |
| 64 | 25 | F | 22.8 | Negative | ND | NA |
| 65 | 32 | F | 24.5 | Negative | ND | NA |
| 66 | 30 | F | 58.5 | Negative | ND | NA |
| 67 | 26 | F | 26.1 | Negative | ND | NA |
| 68 | 45 | F | 48.8 | Negative | ND | NA |
| 69 | 24 | M | 47 | Negative | ND | NA |
| 70 | 22 | M | 47.7 | Negative | ND | NA |
| 71 | 45 | M | 17.3 | Negative | ND | NA |
| 72 | 32 | M | 27.9 | Negative | ND | NA |
| 73 | 32 | M | 31.4 | Negative | ND | NA |
| 74 | 45 | M | 22.3 | Negative | ND | NA |
| 75 | 18 | M | 45.5 | Negative | ND | NA |
| 76 | 35 | M | 18.7 | Negative | ND | NA |
| 77 | 18 | M | 18.8 | Negative | ND | NA |
| 78 | 35 | M | 55.2 | Negative | ND | NA |
| 79 | 28 | M | 88.9 | Negative | ND | NA |
| 80 | 44 | M | 31.8 | Negative | ND | NA |

*ND=Not detected; NA=Not applicable*
