# Supplementary material for: Real-time PCR in detection and quantitation of Leishmania donovani for the diagnosis of Visceral Leishmaniasis patients and the monitoring of their response to treatment
Source: PLoS One. 2017 Sep 28;12(9):e0185606. doi: 10.1371/journal.pone.0185606 (PMC5619796; doi:10.1371/journal.pone.0185606)
Supplement: S6 Table — (DOCX) [file pone.0185606.s006.docx]

**Supporting information**

**S6 Table: Result of Ln-PCR and Real time PCR in buffycoat DNA of TB patients.**

| SL | Age | Sex | DNA concentration (ng/µL) | Ln-PCR | Real Time PCR | |
| --- | --- | --- | --- | --- | --- | --- |
|  |  |  |  |  | Ct | Parasite Load |
| 1 | 22 | M | 86.1 | Negative | ND | NA |
| 2 | 38 | F | 55.6 | Negative | ND | NA |
| 3 | 58 | M | 21.7 | Negative | ND | NA |
| 4 | 54 | F | 34.6 | Negative | ND | NA |
| 5 | 23 | M | 73.7 | Negative | ND | NA |
| 6 | 42 | F | 55 | Negative | ND | NA |
| 7 | 26 | M | 44 | Negative | ND | NA |
| 8 | 58 | F | 34.7 | Negative | ND | NA |
| 9 | 44 | F | 50.2 | Negative | ND | NA |
| 10 | 25 | M | 85.8 | Negative | ND | NA |

*ND=Not detected; NA=Not applicable*
